# Transgenerational impact of climatic changes on cotton production

**DOI:** 10.3389/fpls.2023.987514

**Published:** 2023-03-31

**Authors:** Muhammad Awais Farooq, Waqas Shafqat Chattha, Muhammad Sohaib Shafique, Umer Karamat, Javaria Tabusam, Sumer Zulfiqar, Amir Shakeel

**Affiliations:** ^1^ Department of Plant Breeding and Genetics, University of Agriculture, Faisalabad, Pakistan; ^2^ Molecular Virology Laboratory, National Institute of Biotechnology and Genetic Engineering, Faisalabad, Pakistan; ^3^ State Key Laboratory of North China Crop Improvement and Regulation, Key Laboratory of Vegetable Germplasm Innovation and Utilization of Hebei, Collaborative Innovation Center of Vegetable Industry in Hebei, College of Horticulture, Hebei Agricultural University, Beijing, China; ^4^ Institute of Crop Sciences, Chinese Academy of Agricultural Sciences, Beijing, China

**Keywords:** *Gossypium hirsutum* L. (cotton), climate change, high temperature, drought, antioxidants, reactive oxygen species (ROS)

## Abstract

Changing climatic conditions are an increasing threat to cotton production worldwide. There is a need to develop multiple stress-tolerant cotton germplasms that can adapt to a wide range of environments. For this purpose, 30 cotton genotypes were evaluated for two years under drought (D), heat (H), and drought + heat stresses (DH) under field conditions. Results indicated that plant height, number of bolls, boll weight, seed cotton yield, fiber fineness, fiber strength, fiber length, K^+^, K^+^/Na^+^, relative water contents (RWC), chlorophyll a and b, carotenoids, and total soluble proteins got reduced under D and H and were lowest under DH, whereas superoxidase dismutase (SOD), H_2_O_2_, Na^+^, GOT%, total phenolic contents, ascorbate, and flavonoids got increased for consecutive years. Correlation studies indicated that there was a positive correlation between most of the traits, but a negative correlation with H_2_O_2_ and Na^+^ ions. PCA and clustering analysis indicated that MNH-786, KAHKSHAN, CEMB-33, MS-71, FH-142, NIAB-820, CRS-2007, and FH-312 consistently performed better than other genotypes for most traits under stress conditions. Identified genotypes can be utilized in the future cotton breeding program to develop high-yielding, climate change-resilient cotton.

## Introduction

Cotton plays a significant role in the economy of the country. It is the main source of fiber, oil, and feed for the livestock in the country (Salimath et al., 2021). Pakistan ranks fifth among the top cotton-producing countries after India, China, the United States, and Brazil (Fiaz et al., 2021). Due to changing climatic conditions, cotton is facing many abiotic and biotic stresses, which are negatively impacting crop yield. Abiotic stresses, i.e., heat, salt, and drought, are exacerbating a global problem, as these hamper normal plant growth and morphological and physiological development processes that lead to a reduction in crop yield (Abdelraheem et al., 2019).

The search for cotton germplasm resilient to varied abiotic stresses has intensified due to climate change (Ur Rahman et al., 2020). According to the IPCC, the rate of rise in temperature during 2000–2010 has been recorded at 2.2% in comparison to the temperature regime between 1970 and 2000. Moreover, the projected temperature will rise by 2.6–4.8°C from 2016 to 2035 (Jaiswal et al., 2019). Cotton being a C3 plant and a heat-sensitive plant, the yield of cotton is affected by 10%–17%, with the rise of 1°C in temperature (Zafar et al., 2022). High temperatures lead to high evaporation, which results in a high concentration of salts in the rhizosphere that induces salt stress that causes the reduction of water availability to plants (Saleem et al., 2021). Even a short-term water deficit at the boll development stage in cotton can lead to huge yield losses. At the cellular level, drought induces oxidative stress by the overproduction of reactive oxygen species (ROS), which ruptures the cell membrane and stimulates the cascade of oxidative stresses (Jans et al., 2021; Naz et al., 2022). At the plant level, it results in the inhibition of cell division, expansion of leaf surface area, developmental changes, metabolic adaptations, growth of the stem, and proliferation of root cells. In concert, abiotic stresses dramatically reduce the plant’s productivity and might lead to the death of the plant upon prolonged exposure (Ullah et al., 2019).

Abiotic stresses affect plant antioxidant activities, leading to decreased cotton seed yield. Oxidative stress induces ROS such as hydrogen peroxide (H_2_O_2_), superoxide radicals (O_2_^−^), singlet oxygen (^1^O_2_), and hydroxyl radicals (OH^−^) that are produced in high amounts (Miller et al., 2010). The higher production of ROS damages the plant cell organelles such as chloroplasts, peroxisomes, and mitochondria through oxidation (Wang et al., 2019; Munir et al., 2022). To counteract oxidative damage, protect organelles, and maintain the plant’s cellular functions, the cell produces antioxidant enzymes. The detoxifying enzymes that are produced in the cell are superoxidase dismutase (SOD), peroxidases (POD), catalase (CAT), and non-enzymatic antioxidants including carotenoids, flavonoids, and ascorbate. Heat and drought stresses are highly detrimental to the cotton plants, so it is the primary objective of the cotton breeders to develop germplasm that can produce a higher yield under changing climatic conditions (Wang et al., 2017).

To combat these abiotic stresses, numerous approaches have been undertaken to develop resilience in cotton plants (Haque et al., 2018). Cotton breeders consider the development of climate change-resistant germplasm to be the only effective, reliable, and long-lasting solution (Ur Rahman et al., 2020). Exploitation of natural variation present in the available germplasm, such as screening of available germplasm based on morphological, physiological, and biochemical traits, can lead to a significant level of tolerance in cotton plants against abiotic stresses. The measurements of the antioxidant enzymes produced as a result of the onset of abiotic stress can be used as an effective strategy to screen the available germplasm that can produce a higher yield under the changing climatic conditions (Abbas, 2020). Accurate knowledge of the correlation of different traits with each other at the onset of multiple stresses can assist in the development of those combinations of traits that actively participate in the enhancement of seed cotton yield (Farooq et al., 2018). Moreover, principal component analysis can efficiently dissect the trait associations, interactions among the traits, and performance of the genotypes (Farooq et al., 2019b).

For this purpose, a study was designed in which cotton genotypes were evaluated under multiple combinations and at different levels of heat and drought stress for two years with the aim of developing changing climate-tuned cultivars. The objective of the present research is (i) to nominate cotton genotypes that can tolerate abiotic stresses and can also be used in cotton breeding programs, and (ii) to develop a selection criterion based on agro-physiological and biochemical traits for the development of climate-resilient cotton cultivars.

## Materials and methods

### Plant material

A set of 30 cotton genotypes with different genetic backgrounds developed by different breeding stations in Pakistan, such as the Central Cotton Research Institute, Multan, the Cotton Research Station Faisalabad, the Cotton Research Station Multan, the Cotton Research Station Vehari, and the Nuclear Institute of Agriculture and Biology, Faisalabad, were collected and grown in a randomized complete block design on ridges in field conditions for two years during June 2020 and June 2021. Row to row distance was 60 cm and plant to plant distance was 45 cm, and all other agronomic practices were followed uniformly throughout the season.

### Experimental treatments

Plants were grown under four treatments: control, drought stress (D), heat stress (H), and drought + heat stress (DH). The imposition of drought stress was carried out by increasing the interval between irrigation times; for normal irrigation, the interval was kept at two weeks, whereas for drought conditions, the irrigation interval was extended to three weeks. At the flowering stage, high temperature stress was imposed for 12 days in September, which increased the temperature by 5–6°C inside the tunnel that was constructed using plastic sheets and bamboo sticks. The plants inside the tunnel were covered during the daytime, while they remained uncovered during the night. A mercury thermometer was used to measure the temperature inside the tunnel.

### Data collection

#### Agronomic traits

Plant height was taken from the first cotyledonary node to the apical bud with a measuring tape when growth halted. Effective mature bolls were counted from all the picks, and their records were maintained for each plant separately. Seed cotton was picked from five plants, and afterwards, their weight was measured on the electronic weighing scale for each genotype. The individual weight of each boll was measured by dividing the total weight of seed cotton picked by the number of bolls picked.

#### Fiber quality traits

A single roller ginning machine (Testex, Model: TB510C, USA) was used to gin the representative sample of seed cotton and it was weighed before ginning. The seeds from each genotype were separated from the lint and GOT (ginning out turn) was calculated by dividing the weight of lint in a sample by the seed cotton weight of the sample, which was expressed in percentage. Lint was further processed to take out the parameters of fiber fineness, fiber strength, and fiber length with a high volume instrument (HVI-900, USTER, USA) (Ibrahim et al., 2021).

#### Ionic analysis

To calculate concentrations of Na^+^ and K^+^ ions, fresh green leaves were harvested from plants at noon, when they reached their state of vegetative maturity. Leaves were dried in a hot air dryer for 72 h and then ground using a pestle and mortar. Leaves were then digested in a sulfuric acid and nitric acid mixture (a molar ratio of 1:2) on a hot plate. On completion of digestion, the material was brought to room temperature by cooling, and analysis was performed on a flame photometer (410 Flame Photometer, Sherwood, UK). The K^+^ to Na^+^ ratio was calculated by dividing K^+^ concentration by Na^+^ concentration.

#### Biochemical traits

For sampling, the top four fully expanded leaf was taken for analyses carried out by the method given by Song et al. (2014). Approximately 0.5 g of cotton samples were taken for enzyme extraction; the leaves were cut with the help of a leaf pincher and then crushed and ground into 1–2 ml of cold potassium phosphate buffer (pH 7.8). The mixture was prepared for 5 min at 1,400 rpm. Residues were discarded, and the supernatant was collected for the determination of biochemical attributes *via* UV spectrophotometers (Evolution One Plus, Thermo Fisher Scientific) at different wavelengths (Sarwar et al., 2017).

#### Hydrogen peroxide (µmol/g-FW)

The Velikova protocol was followed for the determination of H_2_O_2_ (Velikova et al., 2000). Fresh leaf tissues (0.5 g) were blended using tichloroacetic acid (TCA). Approximately 5 ml of a 0.1% (w/v) solution) and then centrifuged at 12,000 rpm for 12 min. The supernatant was collected in a volume of 0.5 ml, and then 0.5 ml of phosphate buffer (pH 7.0) and 1 ml of potassium iodide were added. At the 390 nm wavelength of the UV spectrophotometer, the absorbance capacity of each sample was recorded.

#### Catalase (U/mg)

Enzyme extract (0.1 ml) was mixed with 3 ml of the reaction mixture, which contained 5.9 mM H_2_O_2_ and 50 mM potassium phosphate bufferat a pH of 7.0. CAT activity was recorded with a spectrophotometer at 240 nm wavelength (Liu et al., 2009).

#### Peroxidase (U/mg)

POD solution contained 50 mM phosphate buffer (pH 5), 40 mM H_2_O_2_, 20 mM guaiacol, and 0.1 ml of enzyme extract (Liu et al., 2009). Measurements were taken at 470 nm with an absorbance spectrophotometer.

#### Superoxidase dismutase (U/mg)

The reaction mixture consisted of potassium phosphate buffer (pH 5) + 100 μl NBT + 200 μl of Triton X + 200 μl of methionine + enzyme extracts. Approximately 100 μl were dissolved in 800 μl of distilled water and placed for 15 min under ultraviolet light, and then riboflavin in a quantity of 100 μl was added. The absorbance readings were taken at 560 nm using a spectrophotometer.

#### Total soluble proteins (mg/g-FW)

For protein content measurements, the Bradford reagent method was used. Approximately 100 µl of aliquots were blended with 5 ml of Bradford reagent, and with a spectrophotometer at 595 nm wavelength, the absorbance was recorded (Bradford, 1976).

#### Chlorophyll contents and carotenoids assay

For the determination of carotenoids and chlorophyll a and b, the Arnon method (Arnon, 1949) was followed. Approximately 0.50 g of cotton leaf sample was crushed in 8–10 ml of 80% acetone (v/v). For homogenization, filter paper was used. At 645 and 663 nm, the final solution absorbance value was recorded using a spectrophotometer. The chlorophyll a and b and carotenoids were evaluated as follows.


$$\begin{array}{l} Chl\;a\;(mg\;g^{-1}FW)=[12.7(OD\;663)-2.69(OD\;645)]\;\times \;V/1000\;\times W\\ Chl\;a\;(mg\;g^{-1}FW)=[22.9(OD\;645)-2.69(OD\;663)]\;\times \;V/1000\;\times W \end{array}$$

$$\text{Carotenoids}\;\left(\text{mg}/\text{g}\;\text{FW}\right)\;=\;{\text{A}}^{\text{car}}/\;\text{Em}\;\times \;100$$

$${\text{A}}^{\text{car}}=\;\text{O}.\text{D}\;480\;+\;0.114\;\left(\text{O}.\text{D}\;663\right)-0.638\;\left(\text{O}.\text{D}\;645\right)$$

where,

W = weight of leaf sample, V = volume of sample, and Em = 2,500.

#### Ascorbic acid (ASA mg g^−1^ FW)

For the determination of ascorbic acid, the 2,6-dichloroindophenol (DCIP) method was adopted, as explained by Davies and Masten (1991). For a concise description, each molecule of vitamin C converted a DCIP molecule into a DCIPH2 molecule, and the absorbance was recorded at 520 nm.

#### Total phenolic contents and flavonoids (mg g^−1^ FW)

Total phenolic contents were measured according to the method of Ainsworth and Gillespie (2007), and flavonoid contents were measured according to the method outlined by Zhishen et al. (1999).

#### Leaf relative water contents

Leaf samples for relative water contents were collected at pre-dawn by following the method of Silveira et al. (2009). The RWC% was calculated with a minor modification of Weatherly (1950). The 500-g leaf sample (FW) was immersed overnight in distilled water to get leaf turgidity, then leaves were weighed for turgid weight (TW). To get dry weight (DW), the leaves were oven-dried at 80°C for 24 h. The relative water contents were calculated using the formula given below:


$$\text{RWC\%}=\frac{\left(\text{FW}-\text{DW}\right)}{\left(\text{TW}-\text{DW}\right)}\;\times 100$$

#### Malondialdehyde (MDA nmol g^−1^ FW)

To determine the MDA content in cotton leaves, the method of Cakmak and Horst (1991) was adopted. The 500 mg leaf sample was homogenized in 10 ml of a 0.1% trichloroacetic acid (TCA) solution and centrifuged at 14,000×*g* for 5 min. For each ml of extract, 4.5 ml of thiobarbituric acid (0.5%) was used, and the reaction mixture was heated at 95°C for 30 min and then quickly cooled on an ice bath and centrifuged again at 14,000×*g* for 10 min. The absorbance was calculated by:


$$\;\text{MDA level }\left(\text{nmol}\right)=\frac{∆\left(\text{A}532\text{ nm}-\text{A}600\text{ nm}\right)}{156\;\times \;10^{5}}$$

A = Absorption coefficient with the value of 156 mm^−1^ cm^−1^.

### Statistical analysis

An analysis of variance (ANOVA) was performed with a two-factor randomized complete block design (Steel et al., 1997). Mean data were analyzed for principal components, heat maps, and correlation analyses using the statistical packages “prcomp,” “ggplot2,” and “Hmisc” in R 4.1.1 statistical software. Estimates of heritability analyses were carried out by the protocol outlined by Falconer and Mackay (Falconer, 1996; Racine, 2012).

## Results

### Assessment of variation among cotton genotypes under different treatments

The mean squares from the two-factor analysis of variance showed significant differences for genotypes, treatments, and genotype × treatment interaction for all the agro-physiological, biochemical, and fiber quality traits in both years (**Tables 1**, **2**). As all the traits showed significant differences between genotypes × treatments, the data were analyzed separately for each of the four treatments, i.e., control, D, H, and DH.

**Table 1:** First year mean square values for various agro-physiological, biochemical, and fiber quality traits of 30 cotton genotypes under four treatments.

SOV	Genotypes	Treatments	Replication	Treatments * Genotypes	Error
Traits					
Degree of freedom	29	3	2	87	238
ASA	907**	37,145.4**	2,094.8	13.8	59.9
BW	1.1989**	12.5525**	0.5697	0.05	0.08
CAT	366.18**	3,086.05**	67.21	14.79	14.16
Car	0.01632**	0.06983**	0.02017	0.001	0.002
Chla	0.31028**	0.63965*	0.09242	0.08	0.06
Chlb	0.04768**	0.1087**	0.07183	0.000	0.002
FF	2.20056**	8.39549**	0.90255	0.05	0.103
FL	28.5**	375.58**	9.749	2.14	2.204
FLV	6,066.61**	4,685.76**	7,178.77	0.57	299.29
FS	46.499*	439.118**	16.15	0.87	2.20
GOT	97.952**	248.643*	156.093	0.52	5.02
H202	0.04595*	0.58118**	0.12092	0.002	0.005
K	646.2**	29,019.1**	1,291.5	139.6*	76.7
KNA	3.015*	101.874**	4.857	0.53**	0.206
MDA	0.41053**	1.27567**	1.19759	0.006	0.05
Na	329.9**	10,337.3**	702.3	46.1*	33.2
Nb	7.928**	442.121**	5.278	2.87*	33.2
PH	148.7**	38,409**	859.2	4.6	12.6
POD	37.2949*	54.1865**	61.4598	4.09*	2.44
RWC	375.12*	9,769.94**	684.3	28.82	33.28
SCY	86.01**	1,267.05**	19.76	3.63	6.10
SOD	35.14**	1,187.99**	48.35	6.56	3.05
TPC	4.549**	183.397**	8.384	0.202	0.27
TSP	0.0795**	0.13087**	0.01887	0.001	0.003

ASA, ascorbic acid; BW, boll weight; CAR, carotenoids; CAT, catalase; Chla, Chlorophyll a; Chlb, Chlorophyll b; FF, fiber fineness; FL, fiber length; FLV, flavonoids; FS, fiber strength; GOT, ginning out turn percentage; H_2_0_2_, Hydrogen per oxide; K+, potassium content; K+/Na+, the potassium over sodium ratio; MDA, malondialdehyde; Na^+^, the sodium contents; NBP, Number of bolls per plant; PH, plant height; POD, peroxidase; RWC, Relative water content; SCY, seed cotton yield; SOD, superoxide dismutase; TPC, total phenolic content; TSP, total soluble protein.*, Significant at 1% level; **, Significant at 5% level.

**Table 2:** Second year mean square values for various agro-physiological, biochemical, and fiber quality traits of 30 cotton genotypes under four treatments.

SOV	Genotypes	Treatments	Replication	Treatments * Genotypes	Error
Traits					
DoF	29	3	2	87	238
ASA	932.9**	37,950.7**	3,595.9	17	59.3
BW	1.08**	12.84**	0.13	0.046	0.112
CAT	285.4**	3,044.44**	61.77	29.99**	13.61
Car	0.16**	0.07**	0.008	0.001	0.002
Chla	0.303**	0.54*	0.09	0.07	0.06
Chlb	0.04**	0.10**	0.03	0.000	0.002
FF	2.17**	7.49**	4.43	0.05	0.128
FL	29.36**	382.04**	55.22	2.23	2.25
FLV	6,071.0**	28,511.8**	9,403.9	17.1	294.0
FS	45.55	570.3	30.85	1.15	3.82
GOT	101.19	1,247.33	145.77	0.45	6.04
H202	0.046	0.594	0.12	0.002	0.005
K	667.0	32,020.4	2,636.3	149.1*	105.9
KNA	2.72	110.93	5.37	0.47**	0.19
MDA	0.386	1.44	1.25	0.007	0.046
Na	342.9	10,888.7	740.2	48.1*	35.1
Nb	13.04	549.47	84.76	3.13	3.20
PH	162.1**	33,498.6**	1,637.2	4.2	25.4
POD	25.22**	51.37*	42.3	0.44	2.07
RWC	373.2**	1,4751.6**	1,622.7	24.3	28.3
SCY	89.07**	1,230.16**	29.97	3.66	3.12
SOD	36.03**	1,245.10**	53.01	6.77**	3.21
TPC	4.46**	181.6**	3.45	0.19	0.26
TSP	0.08**	0.13**	0.02	0.001	0.003

ASA, ascorbic acid; BW, boll weight; CAR, carotenoids; CAT, catalase; Chla, Chlorophyll a; Chlb, Chlorophyll b; FF, fiber fineness; FL, fiber length; FLV, flavonoids; FS, fiber strength; GOT, ginning out turn percentage; H_2_0_2_, Hydrogen peroxide; K+, potassium content; K+/Na+, the potassium over sodium ratio; MDA, malondialdehyde; Na^+^, the sodium contents; NBP, Number of bolls per plant; PH, plant height; POD, peroxidase; RWC, Relative water content; SCY, seed cotton yield; SOD, superoxide dismutase; TPC, total phenolic content; TSP, total soluble protein.*, Significant at 1% level; **, Significant at 5% level.

### Heat map analysis under different treatments

**Figure 1:**
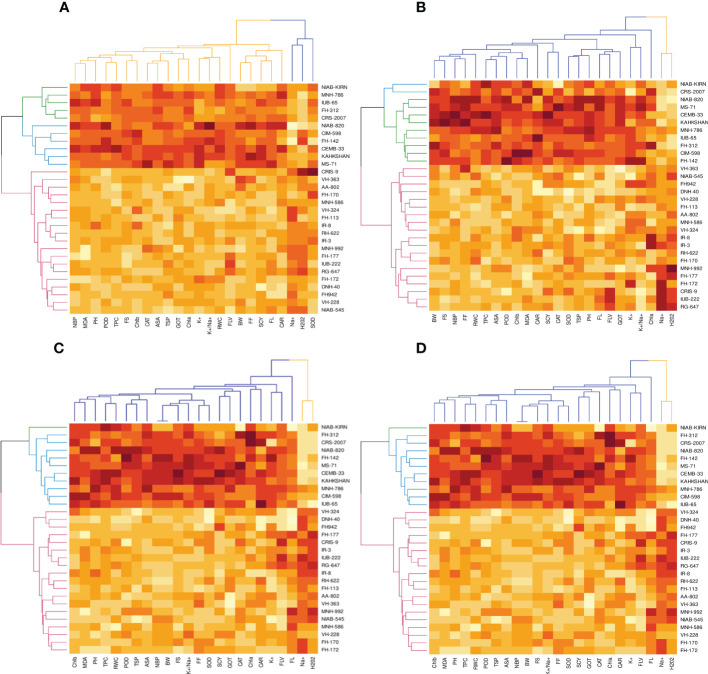
First year PCA-Biplot of various agro-physiological, biochemical and fiber quality traits of 30 cotton genotypes. **(A)** control, **(B)** drought stress **(D)**, **(C)** heat stress (H), and **(D)** drought + heat stress (DH). ASA, ascorbic acid; BW, boll weight; CAR, carotenoids; CAT, catalase; Chla, Chlorophyll a; Chlb, Chlorophyll b; FF, fiber fineness; FL, fiber length; FLV, flavonoids; FS, fiber strength; GOT, ginning out turn percentage; H202, Hydrogen peroxide; K+, potassium content; K+/Na+, the potassium over sodium ratio; MDA, malondialdehyde; Na+, the sodium contents; NBP, Number of bolls per plant; PH, plant height; POD, peroxidase; RWC, Relative water content; SCY, seed cotton yield; SOD, superoxide dismutase; TPC, total phenolic content; TSP, total soluble protein.

**Figure 2:**
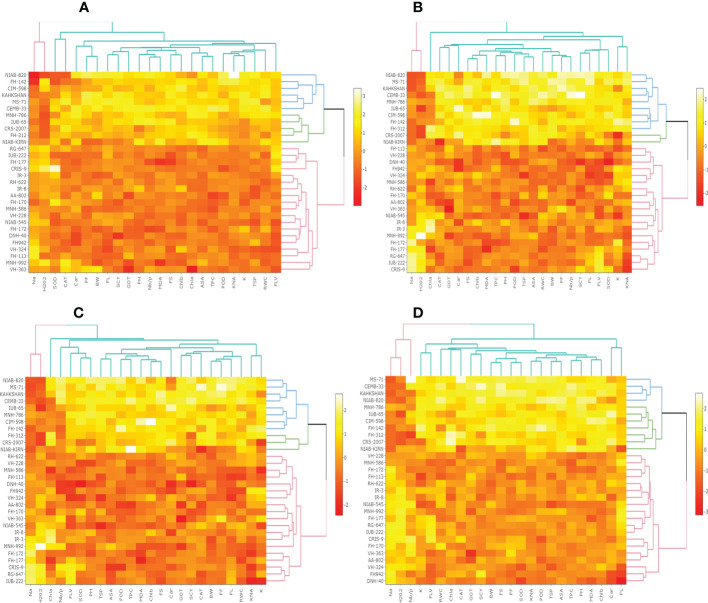
Second year PCA-Biplot of various agro-physiological, biochemical, and fiber quality traits of 30 cotton genotypes **(A)** control, **(B)** drought stress **(D)**, **(C)** heat stress (H), and **(D)** drought + heat stress (DH). ASA, ascorbic acid; BW, boll weight; CAR, carotenoids; CAT, catalase; Chla, Chlorophyll a; Chlb, Chlorophyll b; FF, fiber fineness; FL, fiber length; FLV, flavonoids; FS, fiber strength; GOT, ginning out turn percentage; H202, Hydrogen peroxide; K+, potassium content; K+/Na+, the potassium over sodium ratio; MDA, malondialdehyde; Na+, the sodium contents; NBP, Number of bolls per plant; PH, plant height; POD, peroxidase; RWC, Relative water content; SCY, seed cotton yield; SOD, superoxide dismutase; TPC, total phenolic content; TSP, total soluble protein.

The heat map analysis for control classified the 30 cotton genotypes into five clusters in the first year and four clusters in the second year based on twenty-four agro-physiological and biochemical traits. In the first year, the six cotton genotypes—Niab-Kiran, MNH-786, IUB-65, FH-312, and CRS-2007—were classified in cluster I. It means that these cotton genotypes attained higher values for K^+^, K^+^/Na^+^, POD, CAT, TSP, Chla, Chlb, CAR, MDA, TPC, ASA, RWC, FLV, GOT, FS, FL, PH, BW, NBP, and SCY while lower values for Na^+^, H_2_O_2_, SOD, and FF (**Figure 1**). In the second year, NIAB-820, FH-142, CIM-598, KAHKSHAN, MS-71, and CEMB-33 were grouped into Cluster I. These genotypes were high performers for FF, BW, FL, SCY, GOT, PH, Nb/p, MDA, FS, CHlb, CHla, ASA, TPC, POD, K^+^/Na^+^, K^+^, TSP, RWC, and FLV; and they were low performers for Na^+^, H_2_O_2_, SOD, CAT, and Car (**Figure 2**).

Under drought stress, the 30 cotton genotypes were grouped into four major clusters in both years. In the first year, the six cotton genotypes, viz., Niab-Kiran, CRS-2007, NIAB-820, MS-71, CEMB-33, and KAHKASHAN, attained the highest values for K^+^, K^+^/Na^+^, POD, CAT, TSP, Chla, Chlb, CAR, MDA, TPC, ASA, RWC, FLV, GOT, FS, FL, PH, BW, NBP, and SCY, while the lowest values for Na^+^, H_2_O_2_, SOD, and FF. In the second year, cluster I included seven genotypes, viz., NIAB-820, MS-71, KAHKSHAN, CEMB-33, MNH-786, IUB-65, and CIM-598, which had the highest values for CAT, Car, FS, Chlb, MDA, TPC, PH, POD, TSP, ASA, RWC, BW, FF, Nb/p, SCY, FL, FLV, SOD, K^+^, and K^+^/Na^+^ and had the lowest values for Na^+^, H_2_O_2_, and Chla.

In contrast, under heat stress, the heat map analysis classified the 30 cotton genotypes into two major groups in the first year and six clusters in the second year. In the first year, Group-I consisted of cotton genotypes, viz., IUB-65, CIM-598, MNH-786, KAHKSHAN, CEMB-33, MS-71, FH-142, NIAB-820, CRS-2007, FH-312, and NIAB-KIRN. These cotton genotypes attained comparatively higher values for K+, K+/Na+, POD, CAT, TSP, Chla, Chlb, CAR, MDA, TPC, ASA, RWC, FLV, GOT, FS, FL, PH, BW, NBP, and SCY. In contrast, these values for Na^+^, H_2_O_2_, SOD, and FF are lower. In the second year, NIAB-820, MS-71, KAHKSHAN, and CEMB-33 had high values for all the studied traits except Na^+^, H_2_O_2_, and Chla.

Under DH, the heat map analysis grouped the cotton genotypes into four clusters in the first year and two clusters in the second year. In the first year, for DH, Clusters I, II, and III possessed the highest values for K+, K+/Na+, POD, CAT, TSP, Chla, Chlb, CAR, MDA, TPC, ASA, RWC, FLV, GOT, FS, FL, PH, BW, NBP, and SCY and the lowest values for Na+, H_2_O_2_, SOD, and FF. The cotton genotypes in these three clusters were IUB-65, CIM-598, MNH-786, KAHKSHAN, CEMB-33, MS-71, FH-142, NIAB-820, CRS-2007, FH-312, and NIAB-KIRN. In the second year, MS-71, CEMB-33, KAHKSHAN, and NIAB-820 were grouped into Cluster I and were high performers for all traits except Na^+^, H_2_O_2_, and Nb/p.

### Principal component analysis for different treatments

**Figure 3:**
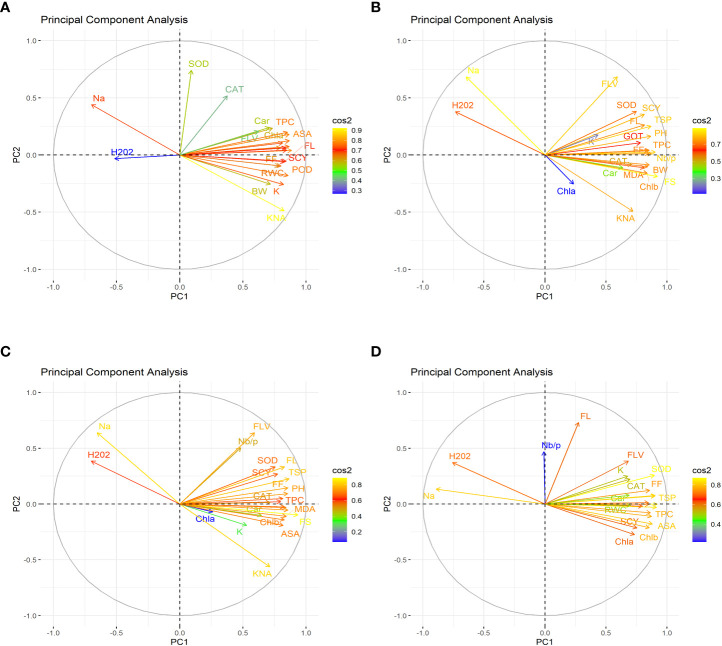
First year heat map analysis based on mean values of various agro-physiological, biochemical, and fiber quality traits of 30 cotton genotypes **(A)** control, **(B)** drought stress **(D)**, **(C)** heat stress (H), and **(D)** drought + heat stress (DH). ASA, ascorbic acid; BW, boll weight; CAR, carotenoids; CAT, catalase; Chla, Chlorophyll a; Chlb, Chlorophyll b; FF, fiber fineness; FL, fiber length; FLV, flavonoids; FS, fiber strength; GOT, ginning out turn percentage; H202, Hydrogen peroxide; K+, potassium content; K+/Na+, the potassium over sodium ratio; MDA, malondialdehyde; Na+, the sodium contents; NBP, Number of bolls per plant; PH, plant height; POD, peroxidase; RWC, Relative water content; SCY, seed cotton yield; SOD, superoxide dismutase; TPC, total phenolic content; TSP, total soluble protein.

**Figure 4:**
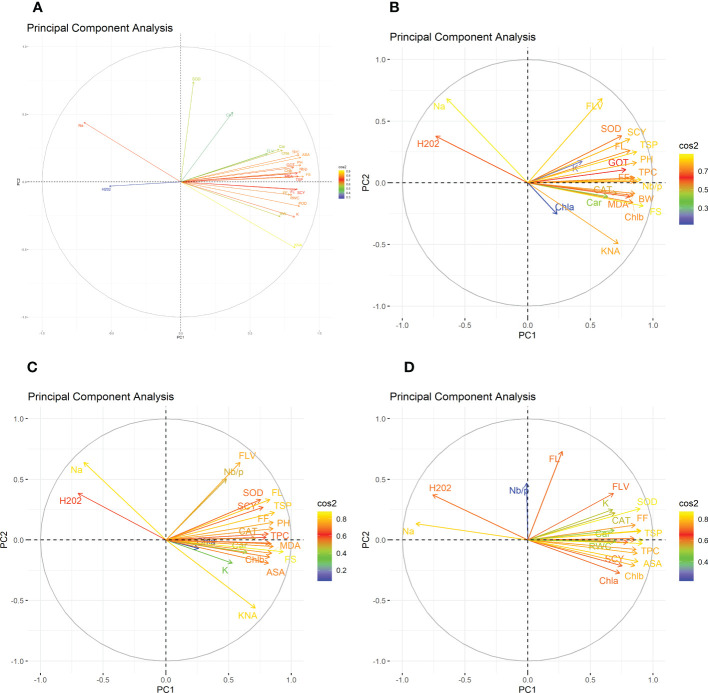
Second year heat map analysis based on mean values of various agro-physiological, biochemical, and fiber quality traits of 30 cotton genotypes **(A)** control, **(B)** drought stress **(D)**, **(C)** heat stress (H), and **(D)** drought + heat stress (DH). ASA, ascorbic acid; BW, boll weight; CAR, carotenoids; CAT, catalase; Chla, Chlorophyll a; Chlb, Chlorophyll b; FF, fiber fineness; FL, fiber length; FLV, flavonoids; FS, fiber strength; GOT, ginning out turn percentage; H202, Hydrogen peroxide; K+, potassium content; K+/Na+, the potassium over sodium ratio; MDA, malondialdehyde; Na+, the sodium contents; NBP, Number of bolls per plant; PH, plant height; POD, peroxidase; RWC, Relative water content; SCY, seed cotton yield; SOD, superoxide dismutase; TPC, total phenolic content; TSP, total soluble protein.

In the first year, under control conditions, the first (PC-1) and second (PC-2) components explained 60.46% and 7.07% of total variation, respectively. PC-1 exhibited positive correlations with H_2_O_2_ and Na^+^. The lowest values for these two traits were desirable, and there were no single cotton genotypes interacting negatively with these two vectors. The PC-2 exhibited positive correlations with K^+/^Na^+^, SOD, POD, CAT, TSP, Chla, Chlb, CAR, MDA, TPC, ASA, RWC, FLV, GOT, FS, FL, PH, NBP, and SCY. There are only four cotton genotypes, viz., NIAN-KIRAN, FH-312, CRS-2007, and CIM-598, that had the highest PC-2 scores and were identified as superior for these traits (**Figure 3**). In the second year, the first (PC-1) and second (PC-2) components were responsible for more than 65% of the variations. PC-1 was negatively associated with all traits except H_2_O_2_ and Na^+^. PC-2 was negatively associated with SOD, CAT, FLV, Car, Chla, TPC, ASA, PH, GOT, Chlb, Nb/p, MDA, TSP, and FS, whereas it was positively associated with the rest of the traits (**Figure 4**).

The PCA for drought stress in the first year revealed that the first component (PC-1) explained 59.81% of the total variation and presented a positive correlation with H_2_O_2_ and Na^+^. The cotton genotypes CRIS-9, RG-647, IUB-222, and FH-177 exhibited the highest PC-1 scores. PC-2 explained 7.94% of the variation and possessed a positive correlation with SOD, POD, CAT, TSP, Chlb, CAR, MDA, TPC, ASA, RWC, FLV, GOT, FS, FL, PH, NBP, and SCY. The cotton genotypes MNH-786, IUB-65, CIM-598, IUB-65, and FH-142 had a positive correlation with PC-2 and were identified as superior for these traits. In the second year, PC1 and PC2 contributed more than 67% of the total variations. It was found that PC1 was negatively associated with FLV, SOD, SCY, FL, TSP, K, GOT, PH, FF, TPC, and Nb/p and positively correlated with the rest of the traits, whereas PC2 positively correlated with Na^+^ and H_2_O_2_ and was negatively correlated with the rest of the traits.

Results from the PCA biplot under heat stress in the first year revealed that the first component (PC-1) explained 53.93% of the variation and presented a positive association with H_2_O_2_, Na^+^, and NBP. The cotton genotypes CRIS-9, RG-647, UB-222, and FH-177 exhibited positive scores for these traits. PC-2 explained 8.58% of the variation and had positive associations with SOD, TSP, Chlb, CAR, MDA, TPC, RWC, FLV, GOT, FS, FL, PH, and SCY. The cotton genotypes IUB-65, MNH-586, and NIAB-KIRN had positive associations with PC-2 and higher values for these traits. In the second year, PC1 and PC2 were responsible for more than 65% of the variations. The PC1 was negatively associated with FLV, Nb/p, SOD, SCY, FL, TSP, FF, PH, CAT, and TPC, whereas the PC2 was positively associated with all the studied traits and was positively associated with Na^+^ and H_2_O_2_.

For the PCA biplot under drought and heat stress in the first year, the first (PC-1) and second (PC-2) components explained 65.62% and 6.03% of the variation, respectively. PC-1 showed a positive association with H_2_O_2_ and Na^+^. The cottons VH-228, AA-802, IR-3, IUB-222, and FH-170 had a positive association with PC-1 and attained the highest values for H_2_O_2_ and Na^+^. Similarly, PC-2 had the positive association with K^+^, K^+^/Na^+^, SOD, POD, TSP, Chla, Chlb, CAR, MDA, TPC, RWC, GOT, FS, PH, and SCY. The cotton genotypes NIAB-KIRN, CIM-598, CRS-2007, FH-312, IUB-65, FH-142, and MNH-786 have the highest values for these traits and interact positively with PC-2. In the second year, PC1 contributed 61.98% and PC2 contributed 6.20% to total variations. PC1 was positively associated with Chla, SCY, Chlb, MDA, ASA, TPC, FS, RWC, and Car, whereas it was negatively associated with the rest of the traits. PC2 was positively associated with Na^+^, H_2_O_2_, and Nb/p and was negatively associated with the rest of the traits.

### Correlation analysis among 24 agro-physiological, biochemical, and fiber quality traits under different treatments

The correlation among different agro-physiological and biochemical traits is of great importance for selecting the most appropriate genotype in a specific environment. The Pearson correlation coefficients were calculated for two years under each of the four conditions separately, *viz*., control, D, H, and DH.

**Figure 5:**
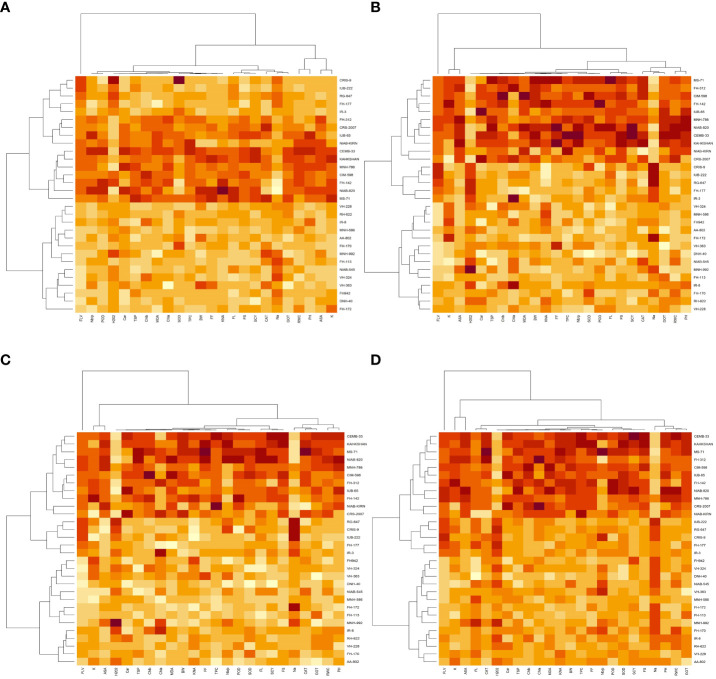
First year correlation analysis based on mean values of various agro-physiological, biochemical, and fiber quality traits of 30 cotton genotypes. **(A)** control, **(B)** drought stress **(D)**, **(C)** heat stress (H), and **(D)** drought + heat stress (DH).ASA, ascorbic acid; BW, boll weight; CAR, carotenoids; CAT, catalase; Chla, Chlorophyll a; Chlb, Chlorophyll b; FF, fiber fineness; FL, fiber length; FLV, flavonoids; FS, fiber strength; GOT, ginning out turn percentage; H202, Hydrogen peroxide; K+, potassium content; K+/Na+, the potassium over sodium ratio; MDA, malondialdehyde; Na+, the sodium contents; NBP, Number of bolls per plant; PH, plant height; POD, peroxidase; RWC, Relative water content; SCY, seed cotton yield; SOD, superoxide dismutase; TPC, total phenolic content; TSP, total soluble protein.

**Figure 6:**
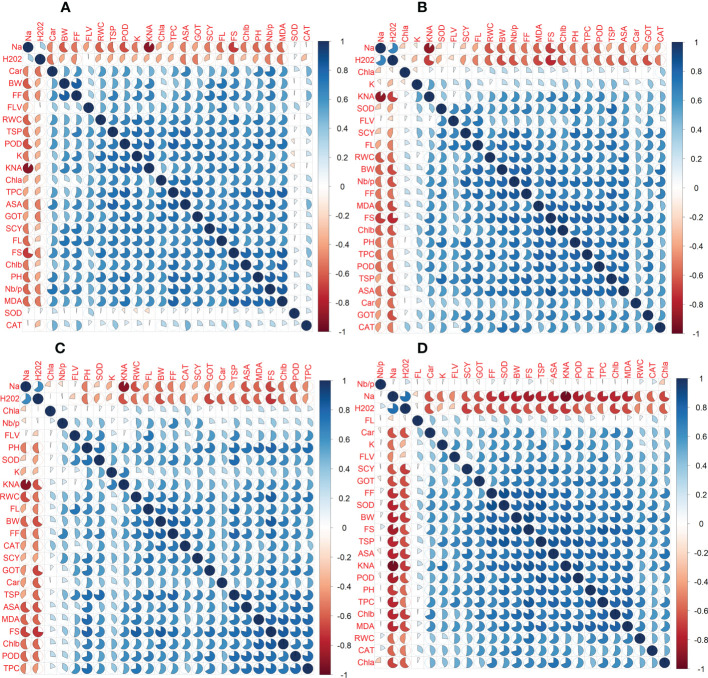
Second year correlation analysis based on mean values of various agro-physiological, biochemical, and fiber quality traits of 30 cotton genotypes. **(A)** control, **(B)** drought stress **(D)**, **(C)** heat stress (H), and **(D)** drought + heat stress (DH). ASA, ascorbic acid; BW, boll weight; CAR, carotenoids; CAT, catalase; Chla, Chlorophyll a; Chlb, Chlorophyll b; FF, fiber fineness; FL, fiber length; FLV, flavonoids; FS, fiber strength; GOT, ginning out turn percentage; H202, Hydrogen peroxide; K+, potassium content; K+/Na+, the potassium over sodium ratio; MDA, malondialdehyde; Na+, the sodium contents; NBP, Number of bolls per plant; PH, plant height; POD, peroxidase; RWC, Relative water content; SCY, seed cotton yield; SOD, superoxide dismutase; TPC, total phenolic content; TSP, total soluble protein.

In the first year, correlation analysis among 24 agro-physiological, biochemical, and fiber quality traits under control revealed that ASA, CAR, and CAT have positive correlations with all the other traits, while Na^+^ and H_2_O_2_ have negative correlations **Figure 5**. The fiber quality traits FS, FF, and FL have also a positive correlation with the majority of agro-physiological and biochemical traits except Na^+^, H_2_O_2_, and SOD, for which there is a negative correlation. Similarly, SCY has a positive but not significant correlation with all agro-physiological and biochemical traits except Na^+^, H_2_O_2_, and SOD. In the second year, most of the traits were positively correlated with each other but were negatively correlated with Na^+^, H_2_O_2_, SOD, and CAT (**Figure 6**).

Under drought stress, in the first year, the ASA, CAR, and CAT have positive correlations with all agro-physiological traits and a negative correlation with Na^+^ and H_2_O_2_. The Na^+^ and H_2_O_2_ had negative associations with all other agro-physiological, biochemical traits, and fiber quality traits. The SCY had also a positive but not significant correlation with all agro-physiological, biochemical, and fiber quality traits except Na^+^, H_2_O_2_, and SOD. In the second year, SCY was positively correlated with K^+^, SOD, FLV, FL, RWC, BW, Nb/p, MDA, TSP, CAT, GOT, Car, PH, and Chla and Chlb, but it was negatively correlated with Na^+^ and H_2_O_2_. Na^+^ and H_2_O_2_ were negatively correlated with all traits.

Under heat stress in the first year, the ASA, CAR, and CAT have positive correlations with all agro-physiological, biochemical, and fiber quality traits, while having a negative correlation with Na^+^ and H_2_O_2_. The Na^+^ and H_2_O_2_ had negative associations with all other agro-physiological, biochemical traits, and fiber quality traits. The SCY also had a positive but not significant correlation with all agro-physiological, biochemical, and fiber quality traits except Na^+^ and H_2_O_2_. In the second year, Na^+^ and H_2_O_2_ were negatively correlated with all the studied traits, whereas all the other traits were positively correlated with each other.

The correlation analysis under DH during the first year among 24 agro-physiological, biochemical, and fiber quality traits predicted that the ASA, CAR, and CAT would have positive correlations with all agro-physiological, biochemical, and fiber quality traits, while having a negative correlation with Na^+^ and H_2_O_2_. The SCY also had a positive but not significant correlation with all agro-physiological, biochemical, and fiber quality traits except Na^+^ and H_2_O_2_. The Na^+^ and H_2_O_2_ had negative association with all other agro-physiological, biochemical, and fiber quality traits. In the second year, it was observed that Nb/p, Na^+^, and H_2_O_2_, were negatively associated with the other traits, whereas the other studied traits are positively associated with each other.

### Coefficient of variation and heritability analysis

During the first year, under the control conditions, most of the traits indicated a lower coefficient of variation (CV) except H_2_O_2_, POD, CAT, TSP, Chla and b, Car, MAD, and TPC; however, with the rise of abiotic stresses, the CV of most of the agro-physiological traits increased except, i.e., K^+^ ions, Na^+^ ions, POD, Chla, and Car. In the second year, most of the traits indicated a rise in CV with the increase in stress level, especially morphological traits; however, the CV was low under heat stress in comparison to drought stress. For heritability analysis, most of the traits showed moderate to high broad-sense heritability in both years; SCY, BW, and fiber traits indicated high heritability (**Tables 3**, **4**).

**Table 3:** First year components of variability and heritability of various traits under control and stress conditions.

SOV	Plant Height					Number of Bolls			Seed Cotton Yield				Boll Weight				Fiber Fineness			
	S_0_	S1	S2	S3	S_0_	S1	S2	S3	S_0_	S1	S2	S3	S_0_	S1	S2	S3	S_0_	S1	S2	S3
Mean	97.76	74.78	65.57	50.6	8.6	6.27	6.5	3.2	24.01	20.48	20.08	14.9	2.9	2.5	2.5	2.01	4.02	3.82	3.78	3.3
Max	113.35	82.69	76.90	57.48	12	10	11	6	33.37	28.65	29.76	22.32	4.2	3.434	3.4	3.13	5.2	4.94	4.94	4.28
Min	90.25	67.41	60.67	45.5	6	3	3	1	16.32	11.65	12.89	9.63	2.27	1.82	1.9	1.12	3.1	2.65	2.62	1.54
CV	4.53	4.41	4.23	4.42	11.42	14.63	13.52	24.89	12.26	13.15	10.46	15.1	9.37	11.08	11.13	14.72	9.57	10.87	11.12	12.38
H^2^	47.98	49.37	49.43	49.17	47.64	31.72	22.48	34.19	58.36	66.87	38.35	57.96	44.74	57.41	59.16	48.75	68.09	71.82	72.17	47.58
SOV	Fiber Strength				Fiber Length				GOT%				K^+^ Ion Concentration				Na^+^ Ion Concentration			
	S_0_	S1	S2	S3	S_0_	S1	S2	S3	S_0_	S1	S2	S3	S_0_	S1	S2	S3	S_0_	S1	S2	S3
Mean	22.46	19.35	19.72	17.08	27.51	24.76	24.72	22.52	38.57	42.05	41.2	44.62	149.3	128.31	128.15	105.34	33.65	43.06	43.21	59.49
Max	26.51	24.65	26.62	22.43	32.36	29.44	31.39	24.96	45.71	49.83	48.46	51.52	183.6	150.8	159.84	128.21	42.23	60.51	61.11	78.65
Min	16.65	15	15.15	12.52	20.25	18.02	19.65	19.33	29.77	32.45	31.55	33.42	126.4	106.14	105.45	84.909	21.63	33.37	31.34	37.01
CV	8.39	9.46	9.5	10.85	6.55	6.55	6.5	4.18	6.17	6.17	6.172	6.1	9.16	3.6	4.05	4.72	9.76	9.2	9.6	13.95
H^2^	64.88	68.22	67.7	59.56	60.18	60.3	59.02	49.06	56.09	56.06	56.25	55.91	81.41	21.66	20.94	29.33	50.08	34.63	33.31	56.16
SOV	K^+^/Na^+^ ratio				Hydrogen Peroxide				Superoxidase dismutase				Peroxidase dismutase				Catalase			
	S_0_	S1	S2	S3	S_0_	S1	S2	S3	S_0_	S1	S2	S3	S_0_	S1	S2	S3	S_0_	S1	S2	S3
Mean	4.45	3.07	3.06	1.85	0.14	0.22	0.23	0.344	6.01	9.72	10.37	14.87	11.03	9.98	9.98	9.23	42.84	36.74	36.78	28.7
Max	8.06	4.71	5.1	3.05	0.31	0.46	0.488	0.707	9.57	13.72	14.6	22.65	18.69	14.73	14.73	13.99	55.58	49.96	53.15	44.74
Min	2.8	2.05	1.99	1.11	0.01	0.05	0.05	0.07	4.15	5.57	5.91	6.69	7.24	6.22	6.23	5.48	32.32	21.32	23.04	17.26
CV	21.606	10.42	11.36	18.46	22.63	22.22	20.41	20.35	9.68	12.32	12.32	22.06	18.96	16.74	16.74	17.24	12.77	14.58	15.79	14.77
H^2^	80.76	30.8	31.7	56.43	32.9	36.1	31.98	33.11	29.42	31.03	31.13	69.66	61.45	61.37	61.37	60.89	67.14	70.7	73.68	57.64
SOV	Total Soluble Proteins				Chlorophyll a				Chlorophyll b				Carotenoids				Malondialdehyde			
	S_0_	S1	S2	S3	S_0_	S1	S2	S3	S_0_	S1	S2	S3	S_0_	S1	S2	S3	S_0_	S1	S2	S3
Mean	0.301	0.25	0.26	0.209	1.001	0.893	0.88	0.79	0.32	0.28	0.27	0.24	0.22	0.22	0.22	0.17	0.86	1.01	1.04	1.16
Max	0.53	0.47	0.48	0.41	1.83	1.58	1.56	2.006	0.66	0.6006	0.58	0.487	0.36	0.34	0.36	0.27	1.3	1.99	1.97	2.19
Min	0.11	0.01	0.02	0.017	0.136	0.24	0.24	0.199	0.14	0.129	0.12	0.11	0.101	0.103	0.10	0.07	0.12	0.54	0.53	0.63
CV	23.27	30.34	30.32	34.007	22.05	16.18	16.41	16.61	17.98	18.8	19.89	24.11	18.05	12.39	12.34	12.32	11.57	12.75	12.62	13.75
H^2^	59.06	56.08	56.87	63.96	47.19	27.65	28.15	21.9	49.67	50.66	54.35	70.89	41.69	23.7	22.77	22.65	22.69	25.7	26.17	31.41
SOV	Total Phenolic Contents				Ascorbic Acid				Relative Water Contents				Flavonoids							
	S_0_	S1	S2	S3	S_0_	S1	S2	S3	S_0_	S1	S2	S3	S_0_	S1	S2	S3	S_0_	S1	S2	S3
Mean	2.89	4.02	4.16	6.31	133.89	148.82	149.84	182.11	72.83	58.44	57.17	41.63	322.51	353.7	353.73	364.77				
Max	4.01	5.69	6.89	8.59	153	167.9	182.58	206.93	89.38	74.76	72.96	59.07	375	408.307	408.01	419				
Min	1.54	2.66	2.29	4.25	120.39	130.02	117.28	156.03	51.24	42.86	41.96	29.39	276	307.08	307.03	318.02				
CV	12.63	11.55	14.34	12.94	4.7	4.8	5.9	4.8	5.009	11.34	10.7	9.9	5.87	5.67	5.67	5.5				
H^2^	53.98	55.01	55.13	58.1	55.25	59.37	48.82	59.52	27.73	69.46	58.07	38.39	55.47	57.26	57.26	57.26				

**Table 4:** Second year components of variability and heritability of various traits under control and stress conditions.

SOV	Plant Height					Number of Bolls			Seed Cotton Yield				Boll Weight				Fiber Fineness			
	S_0_	S1	S2	S3	S_0_	S1	S2	S3	S_0_	S1	S2	S3	S_0_	S1	S2	S3	S_0_	S1	S2	S3
Mean	96.49	72.87	67.42	49.74	9.97	7.56	6.48	4.02	23.98	20.39	20.07	14.99	2.91	2.37	2.49	1.99	3.96	3.71	3.70	3.27
Max	115.62	85.99	79.2	58.36	13	11	13	8	32.7	30.25	29.03	22.23	4.36	3.32	3.51	3.16	5.4	4.98	5.13	4.19
Min	87.54	63.37	59.46	41.72	7	4	2	1	15.9	11.42	12.63	9.34	2.27	1.67	1.84	1.07	3.06	2.49	2.59	1.5
CV	4.5	4.38	4.38	4.45	9.89	12.63	24.31	17.84	12.26	13.6	10.44	15.61	9.37	11.34	11.18	14.69	9.86	10.84	11.37	12.27
H^2^	47.57	49.09	49.26	49.06	47.64	34.7	39.95	29.46	58.92	64.82	37.44	58.72	44.24	56.55	58.97	48.45	69.55	72.22	71.19	47.96
SOV	Fiber Strength				Fiber Length				GOT%				K^+^ Ion Concentration				Na^+^ Ion Concentration			
	S_0_	S1	S2	S3	S_0_	S1	S2	S3	S_0_	S1	S2	S3	S_0_	S1	S2	S3	S_0_	S1	S2	S3
Mean	22.39	18.17	20.09	16.55	27.33	24.52	24.45	22.31	37.68	45.74	41.07	44.39	152	126.12	129.43	105.95	34.55	44.57	44.37	61.08
Max	26.72	25.56	26.88	23.32	32.67	29.40	31.07	25.21	44.8	53.12	47.49	53.07	187.27	149.19	169.44	135.9	43.49	62.32	62.95	81.01
Min	16.32	12.9	15.19	11.61	20.05	17.84	19.45	15.65	29.17	35.37	30.92	32.75	129.01	105.08	103.45	83.21	22.27	34.37	32.28	38.126
CV	8.39	10.38	9.39	11.05	6.55	6.55	6.8	4.84	6.5	5.9	6.16	6.09	9.26	3.65	4.05	4.96	9.7	9.16	9.54	13.78
H^2^	65.09	76.31	67.02	60.33	60.18	60.26	62.92	47.4	60.68	53.88	56.59	56.33	81.73	22.04	20.15	29.92	49.97	34.37	32.95	55.52
SOV	K^+^/Na^+^ ratio				Hydrogen Peroxide				Superoxidase dismutase				Peroxidase dismutase				Catalase			
	S_0_	S1	S2	S3	S_0_	S1	S2	S3	S_0_	S1	S2	S3	S_0_	S1	S2	S3	S_0_	S1	S2	S3
Mean	4.5	2.9	3.02	1.81	0.15	0.22	0.23	0.34	6.06	9.78	10.43	15.12	11.12	10.02	10.03	9.29	42.38	36.07	36.11	28.18
Max	7.98	3.92	5.24	2.9	0.31	0.47	0.47	0.72	9.66	14.03	14.55	22.9	18.87	14.58	14.58	13.85	55.15	48.96	52.09	43.85
Min	3.12	1.97	1.89	1.06	0.01	0.05	0.05	0.07	4.15	5.8	6.02	6.75	7.31	6.34	6.36	5.59	31.64	20.89	22.58	16.92
CV	20.29	9.88	11.24	18.6	24.63	21.16	31.97	19.85	9.65	12.32	12.32	22.03	18.96	17.02	16.64	17.28	13.26	14.48	15.6	14.68
H^2^	83.35	32.93	30.91	56.78	41.16	33.84	20.18	31.96	29.37	30.67	31.02	69.54	61.22	61.59	59.76	62.12	67.81	70.32	73.39	57.36
SOV	Total Soluble Proteins				Chlorophyll a				Chlorophyll b				Carotenoids				Malondialdehyde			
	S_0_	S1	S2	S3	S_0_	S1	S2	S3	S_0_	S1	S2	S3	S_0_	S1	S2	S3	S_0_	S1	S2	S3
Mean	0.304	0.25	0.26	0.21	0.97	0.87	0.88	0.78	0.32	0.28	0.28	0.24	0.22	0.22	0.22	0.17	0.86	1.01	1.05	1.16
Max	0.54	0.48	0.49	0.41	1.81	1.55	1.57	2	0.64	0.58	0.57	0.47	0.36	0.34	0.36	0.27	1.32	1.97	1.99	2.21
Min	0.11	0.02	0.02	0.01	0.13	0.24	0.25	0.19	0.14	0.13	0.13	0.01	0.09	0.1	0.1	0.07	0.11	0.53	0.54	0.62
CV	23.37	29.86	30.4	34.08	22.04	14.91	16.42	16.81	17.99	18.52	19.62	23.42	18.03	12.49	11.85	12.58	11.52	12.62	12.84	14.74
H^2^	59.28	57.36	56.98	64.07	47.06	22.39	28.51	21.69	49.88	51.2	54.03	62.85	41.6	23.69	20.8	23.61	22.57	25.86	27.18	34.22
SOV	Total Phenolic Contents				Ascorbic Acid				Relative Water Contents				Flavonoids							
	S_0_	S1	S2	S3	S_0_	S1	S2	S3	S_0_	S1	S2	S3	S_0_	S1	S2	S3	S_0_	S1	S2	S3
Mean	2.88	4.01	4.12	6.28	134.37	149.22	150.95	183.12	73.11	58.5	57.25	41.78	317.84	345.49	348.04	359.15				
Max	3.9	5.5	6.08	8.76	156.06	170.22	186.25	211.07	90.28	76.14	75.14	59.66	364	398.25	398.2	408				
Min	1.57	2.7	2.3	4.21	120.54	131.32	119.63	157.59	49.7	41.58	40.7	28.69	265	287	294	307.02				
CV	12.67	11.58	13.92	12.99	4.77	4.88	5.97	5.03	5.01	11.52	10.44	9.89	5.9	5.91	6.2	5.4				
H^2^	56.66	54.41	58.31	57.68	55.03	60.55	47.82	65.42	27.9	68.03	56.31	38.73	55.47	58.11	61.63	55.23				

## Discussion

Under changing climatic conditions, abiotic stresses are intensifying and are having a negative impact on the cotton crop. These stresses have become a major obstacle to achieving cotton yield potential by disrupting various morphological, physiological, and metabolic processes (Hassan et al., 2020). The development of high yield cultivars tailored according to changing climatic conditions has become inevitable to sustain the high yield of the cotton crop (Wang et al., 2013). To date, efforts have been focused on mostly single abiotic stress; however, under the dynamic climatic circumstances, the situation has ripened such that more than one stress should be applied in a systematic way to the growing cotton plants (Kirungu et al., 2019) so that the climate-resilient cotton genotypes can be identified and can be used in future breeding programs.

Therefore, in the current study, cotton genotypes were subjected to multiple abiotic stresses in different combinations, i.e., drought, heat, and DH, for two consecutive years. Results indicated that under control, all the genotypes performed well, whereas under drought and heat stresses, all the traits showed negative impact. However, the values of all traits were almost similar for all the traits under these two stresses (Singh et al., 2018; Sabagh et al., 2020). However, when DH was applied, it was observed that there was a highly negative impact on all the cotton genotypes. It was observed that, with the changing climatic conditions, there will be a highly negative impact on the cotton genotypes.

Under these abiotic stresses, morphological traits, i.e., plant height, number of bolls, SCY, and boll weight, reduced under the drought and heat stresses, and the reduction in these traits was more pronounced under the DH stress (Singh et al., 2018). At the onset of stress, it is proposed that plants sense osmotic changes first due to drought and heat stress rather than sodium ions, while sodium-specific responses occur much later through the toxic effects of sodium and chloride ions on the leaves (Munns and Tester, 2008). The plant height was decreased due to the reduction in cell wall elasticity due to the absorption of substances that were produced in response to disturbed metabolic processes triggered by the accumulation of toxic ions inside the cell. The absorption of undesirable metabolites decreased cell expansion, and the reduction in cell wall turgidity caused the shoots to remain shorter (Zafar et al., 2020). The reduction in the number of bolls can be attributed to the shrinkage of cell walls, hindrance in the differentiation of the tissues, disturbance of essential ions, and injuries to the growing tissues. Reduced cell division and reduction in the cell size caused the decline in the size of a leaf expansion that ultimately led to the decreased photosynthates generation inside the cell, which resulted in the lesser number of bolls (Yavari, 2020). Similarly, boll weight also got reduced under abiotic stresses due to the disturbance of various metabolic pathways involved in the production of ATP, leading to lesser ATP synthesis than the control (Farooq et al., 2020). The reduction of boll weight and number of bolls on the cotton plant under drought, heat, and DH led to a low SCY. All the fiber quality traits indicated a reduction in values except GOT%, which can be due to the fact that the decrease in the size of the seed resulted in more GOT% (Chaudhary et al., 2020).

When plants lose water due to high temperatures or water deficit conditions, the concentration of salts increases in the rhizosphere, which decreases the osmotic pressure. To maintain the cell volume and turgor, plants undergo osmotic adjustments by producing organic solutes in high amounts, i.e., proline, sugar alcohols, and sorbitol, by utilizing energy resource, which compromises the plant photosynthetic processes (Flowers et al., 2015). The reduction of photosynthesis can be related to the less production of chlorophyll a and b contents in a reduced leaf area, ultimately affecting the yield potential (Sanchez et al., 2008). At the onset of high temperatures and drought stress, stomata close to avoid the loss of water, and this closure also reduces the amount of CO_2_ available for fixation, although the expected increase in CO_2_ under climatic changes can only partially recover the photosynthetic rate (Cheeseman, 2013). Moreover, there is also an ionic effect of sodium on photosynthesis; the activity of CO_2_ fixing enzymes decreases during salt stress, and interestingly, the tolerance of these enzymes for Na^+^ *in vitro* differs among various genotypes (Bose et al., 2017). Genotypes that accumulated a high concentration of Na^+^ ions and a low concentration of K^+^ ions inside the cell were regarded as tolerant. High concentrations of Na^+^ ions displace Ca^++^ ions in the cell membrane, which reduces the ability of a cell to exclude Na^+^ ions (Peng et al., 2014). The proton motive force necessary for energy production in chloroplasts depends on the close coordination between PH and electropotential changes over thylakoid membranes. Sodium ions disturb this balance because of their positive change and effect on pH. Overall, drought and heat stresses act in a synergistic fashion and disrupt photosynthesis by disturbing the protein motive force and chloroplast function and by interfering with CO_2_ enzymes (Bose et al., 2017). The rise of abiotic stress further exacerbates the situation by decreasing the hydraulic conductivity of roots by 70% (Boursiac et al., 2005). Hydraulic conductivity is facilitated to a large extent by aquaporins, which are pores that facilitate water transport over membranes (Javot et al., 2003; Postaire et al., 2010). However, under stress conditions, both the localization and the activities of aquaporins and downstream sodium signals are greatly altered (Li et al., 2014).

Plants respond to ROS by upregulating antioxidant defensive enzyme activities, i.e., peroxidase (POD), catalase (CAT), and superoxidase dismutase (SOD) (Cui et al., 2021). With the increase of abiotic stresses, H_2_O_2_ production increases, against which catalase is synthesized, and it induces its scavenging activity, which greatly reduces the negative implications of H_2_O_2_. SOD is an efficient intercellular enzymatic antioxidant, and its activity increases in high-yielding genotypes, rendering them tolerant. It catalyzes the dismutation of superoxide into molecular oxygen and H_2_O_2_ and acts as a first line of defense against ROS. POD and CAT metabolize H_2_O_2_ into water and oxygen inside the cytosol and chloroplasts, which safeguards the cell from the toxic effects of H_2_O_2_ (Liu et al., 2021). Total soluble proteins got reduced due to the toxic ion accumulation inside the cell that disintegrated the cell membrane. The concentration of carotenoids also increased, as they are important antioxidants for protecting singlet oxygen (Sharma et al., 2019). Heat and drought stress decrease the relative water content (RWC), which ultimately reduces the plant photosynthesis rate as well; therefore, genotypes that had a higher RWC under the stresses were declared as tolerant genotypes. The genotypes that had a high RWC also had a high boll retention rate and a high SCY, which are associated with high tolerance under a multitude of abiotic stresses (Mammadov et al., 2018; Anwar et al., 2022). Total phenolic contents (TPCs) play a significant role in the protection of plant cell components. TPCs are positively correlated with the antioxidant activity. The potential of phenolics to act as antioxidants is mainly due to their ability to act as hydrogen donors, reducing agents, and quenchers of singlet oxygen. TPCs increased in the tolerant genotypes, which indicates the capacity of the plants to cope with the implications of a multitude of stresses (Zafar et al., 2020). Malondialdehyde content estimation can be used to access membrane damage (Hessini et al., 2022). A higher level of observed MDA is an indication of structural damage due to the increasing stress triggered by high ROS production. Ascorbic acid/ascorbate (ASA) is another non-enzymatic antioxidant whose concentration inside the cytosol and chloroplasts increased with the rise of stresses, and it can protect the photosynthetic machinery of the plant under these stresses. In our study, genotypes that had high ASA concentrations were able to produce higher yields and were regarded as tolerant (Kamal et al., 2017). The flavonoid synthesis increased with the increase in stress; its accumulation under the stress conditions indicated that it provided a better antioxidant capacity and played an important role in the reduction of oxidative damage, which improved the plant’s tolerance ability against these abiotic stresses (Nix et al., 2017).

To select the best genotypes on the basis of morphological, physiological, and biochemical bases, cluster analysis was carried out and a heatmap was drawn (Hummel et al., 2017). Cluster analysis during the two years under the studied stresses showed that the genotypes MNH-786, KAHKSHAN, CEMB-33, MS-71, FH-142, NIAB-820, and CRS-2007 were present in a single cluster of tolerant genotypes based on 24 agro-physiological and biochemical traits and remained stable. PCA analyses revealed that the first two PCs contributed significantly to the total variation under control and stress conditions for agronomic, fiber-related, morphological, and biochemical traits. These traits affirmed the differences among genotypes regarding the studied traits under control and stress conditions, which can be proved useful for future breeding programs regarding the development of climate-resilient cotton genotypes. H_2_O_2_, Na^+^, K^+^, K^+^/Na^+^, SOD, POD, TSP, Chlb, CAR, MDA, TPC, RWC, GOT, FS, PH, and SCY contributed to the first two PCAs under control and stress conditions. In both years, it was observed that Na^+^ and H_2_O_2_ remained positively correlated with PC2 and negatively correlated with PC1. Moreover, it is also necessary to identify the association of various traits with one another under dynamic climatic conditions; therefore, the correlation matrix was employed to understand the dependency of various traits on each other for better phenotypes and improved yield. During both years, SCY indicated a positive correlation with all traits except Na^+^ ions and H_2_O_2_. Most of the traits were positively correlated with each other; however, H_2_O_2_ and Na^+^ were negatively correlated with all other traits. Expected broad-sense heritability was found moderate to high for the studied traits under all levels of stress which indicates that the characters are genetically controlled (Farooq et al., 2019a). CV was low for most of the traits, which indicated that the data was mostly centered around the mean and there was less variation in the data. Heritability estimates did not necessarily increase with the increase in stress; a few traits indicated an increasing trend, while others showed a declining trend. It indicates that there are genes associated with stress that get activated when stress is applied. It can be further argued that hidden genetic variation that was previously unselected could be uncovered when stress is applied (Khokhar et al., 2017).

In a nutshell, genotypes have indicated that under drought and heat stresses, genotypes performed almost in a similar fashion; however, when DH was applied, only the most tolerant genotypes were able to produce yield at the minimum loss. At the onset of abiotic stresses, plant-intrinsic antioxidant defense mechanisms get activated, which try their best to protect the plant’s photosynthetic machinery from the damages of ROS. However, there is still a need to study underlying synergistic mechanisms under these stresses so that a well-adapted ideotype can be developed under the changing climatic conditions.

## Conclusion

Under the changing climatic conditions, it is imperative to analyze the cotton plants adaptation to various levels of abiotic stresses using agro-physiological and biochemical markers. Resilient germplasm must be developed that has a capacity to tolerate environmental fluctuations without adversely affecting its yield. The current study observed that the cotton genotypes MNH-786, KAHKSHAN, CEMB-33, MS-71, FH-142, NIAB-820, CRS-2007, and FH-312 performed up to 65% better than mean values for studied traits under control and stress conditions during both years and can be efficiently employed in future climate change resilient cotton breeding programs.

## Data availability statement

The original contributions presented in the study are included in the article/supplementary materials. Further inquiries can be directed to the corresponding authors.

## Author contributions

MF: Experimentation, manuscript writing and final editing. WC and MSS: Data collection, manuscript writing, visualization of data, and statistical analysis. UK, SZ. and JT: Formal analysis, data visualization, and validation. AS: Conceptualization, final editing, and approval of the final draft for publication. All authors contributed to the article and approved the submitted version.
